# Educational interventions in child development and health literacy assumptions: an integrative review

**DOI:** 10.1590/0034-7167-2022-0116

**Published:** 2022-12-16

**Authors:** Rayara Medeiros Duarte Luz, Dayana Cecília de Brito Marinho, Ana Paula Esmeraldo Lima, Maria Wanderleya Lavor Coriolano-Marinus

**Affiliations:** I Universidade Federal de Pernambuco. Recife, Pernambuco, Brazil

**Keywords:** Health Education, Health Literacy, Child Development, Health Personnel, Primary Health Care, Educación en Salud, Alfabetización en Salud, Desarrollo Infantil, Personal de Salud, Atención Primaria de Salud, Educação em Saúde, Letramento em Saúde, Desenvolvimento Infantil, Profissionais de Saúde, Atenção Primária à Saúde

## Abstract

**Objectives:**

to analyze scientific evidence in the literature that addresses educational interventions conducted by health professionals on early childhood development in a community context and to identify which health literacy assumptions are present during the implementation of interventions.

**Method:**

an integrative review in PubMed, CINAHL and Web of Science databases. Of 300 studies found, we selected 11 for the sample.

**Results:**

health professionals are trained to implement interventions with parents/caregivers to promote child development in community settings. Parents are encouraged to develop an environment that is encouraging and conducive to the development of their children. The main dimensions of health literacy found were access and apply.

**Conclusion:**

it confirms the importance of training health professionals, with skills and communicative skills to guide parents/caregivers to encourage the development of their children in their family environment with playful and interactive activities.

## RESUMO

**Objetivos:**

analisar na literatura evidências científicas que abordem intervenções educativas conduzidas por profissionais de saúde sobre desenvolvimento infantil na primeira infância em contexto comunitário e identificar quais pressupostos do letramento em saúde estão presentes durante a implementação das intervenções.

**Método:**

revisão integrativa nas bases de dados PubMed, CINAHL e *Web of Science*. De 300 estudos encontrados, 11 foram selecionados para a amostra.

**Resultados:**

os profissionais de saúde são capacitados para implementar intervenções com pais/mães/cuidadores, para promoção do desenvolvimento infantil em contextos comunitários. Os pais são orientados a desenvolver um ambiente estimulante e propício para o desenvolvimento dos filhos. As principais dimensões do letramento em saúde encontradas foram acessar e aplicar.

**Conclusão:**

ratifica-se a importância da capacitação dos profissionais de saúde, com competências e habilidades comunicativas para orientar pais/mães/cuidadores a estimularem o desenvolvimento dos filhos em seu ambiente familiar com atividades lúdicas e interativas.

## RESUMEN

**Objetivos:**

analizar la evidencia científica en la literatura que aborda intervenciones educativas realizadas por profesionales de la salud sobre el desarrollo infantil temprano en un contexto comunitario e identificar qué supuestos de alfabetización en salud están presentes durante la implementación de las intervenciones.

**Método:**

revisión integradora en las bases de datos PubMed, CINAHL y Web of Science. De 300 estudios encontrados, 11 fueron seleccionados para la muestra.

**Resultados:**

los profesionales de la salud están capacitados para implementar intervenciones con padres/madres/cuidadores para promover el desarrollo infantil en entornos comunitarios. Se alienta a los padres a desarrollar un entorno estimulante y propicio para el desarrollo de sus hijos. Las principales dimensiones de alfabetización en salud encontradas fueron acceso y aplicación.

**Conclusión:**

se confirma la importancia de formar profesionales de la salud, con competencias y habilidades comunicativas para orientar a los padres/madres/cuidadores a incentivar el desarrollo de sus hijos en su entorno familiar con actividades lúdicas e interactivas.

## INTRODUCTION

Early childhood is defined as the first six years of a child’s life. During this period, there are numerous changes and adaptations, with accelerated processes in growth and development. Public policies and guidelines focused on children’s health, nationally and internationally, recommend the surveillance of child development (CD) as a priority, which should include health care, guidance to caregivers, early identification and diagnosis of possible delays, interventions for preventive care^(1-2)^.

The Nurturing Care Framework, launched by the World Health Organization (WHO) in 2018, as a set of global actions aimed at responsive care for children in early childhood, highlights CD as a social and political aspect to achieve the Sustainable Development Goals (SDG-2030). CD theme encompasses objectives such as eradicating poverty, ending hunger and improving nutrition, ensuring a healthy life, ensuring quality education and promoting peaceful societies^(3)^.

In these actions, the role of health professionals (HP), together with other sectors allied to the Nurturing Care Framework, should be purposeful in terms of good health, adequate nutrition, responsiveness of caregivers, safety/protection and early learning opportunities through community health services^(3)^.

The participation of parents in the care of their children is of paramount importance to provide an environment that is opportune for development. For the establishment of timely responsive care, educational strategies aimed at parents/caregivers need to consider motivational aspects, knowledge and practical skills that strengthen responsive relationships, age-appropriate stimuli, strong affective relationships and positive parent-child interactions, which promote parent/caregiver autonomy in daily activities that favor the development of young children^(4)^.

Considering the need to articulate HP’s knowledge and actions for a positive response from parents/caregivers to care that involves CD promotion for children aged zero to six years, the health literacy (HL) theoretical framework is a construct that can support decision-making during health work, adding cognitive, motivational, affective and practical elements for decision-making that involve behaviors in health management.

HL consists of a person’s ability to access health information, understand it and process it for decision-making in their health and self-care management^(5)^. A low level of parenting HL is closely related to unfavorable outcomes in child health^(6)^; therefore, professionals play a key role in actually achieving good communication during educational interventions and health education (HE) actions.

Four domains are considered in the HL theoretical framework: 1) access; 2) understand; 3) appraise; 4) apply. Access is the ability to seek and obtain health information. Understand is the ability to understand the information accessed. Appraise is the ability to interpret, filter, judge and assess such information. Finally, apply is the ability to communicate and use information in order to better manage and conduct health^(7)^.

The continuing education of HP in the scope of Primary Health Care (PHC) is of paramount importance, to train and integrate the multidisciplinary team in the lines of child health care, with a view to full development. The team must know the child population assisted in its area of coverage to assess and develop educational health interventions, establishing a good relationship and communication with the final target audience^(1)^.

Educational interventions conducted by nurses focused on CD have benefited responsive relationships in the family context, parenting skills, greater child learning, the prevention of child maltreatment, providing opportunities for the relationship between nurses and caregivers^(8)^.

The present study is justified by the importance of HP in supporting caregivers in making a conscious and responsible decision in the care of young children, with educational interventions to guide parents/caregivers to interact with their children so that they can reach their potential during early childhood.

## OBJECTIVE

To analyze in the literature scientific evidence that addresses educational interventions conducted by HP on CD in early childhood in a community context and identify which HL assumptions are present during the implementation of interventions.

## METHOD

This is an integrative review, whose purpose is to synthesize, in a systematic and orderly way, scientific evidence about a particular topic or problem, so that knowledge on the topic is produced, contributing to Evidence-Based Practice (EBP)^(9)^.

Five steps were taken: (1) establishment of the research question; (2) sample selection and definition of databases and eligibility criteria; (3) data extraction from selected studies; (4) analysis of the main results of the studies included in the review and interpretation of the findings; (5) presentation of knowledge synthesis^(9)^.

### Theme identification and research question development

The PICO strategy (Population, Interest, Context and Outcomes)^(10)^ was used to identify the problem and survey the research question: P - HP; I - educational interventions on CD; C - community context; O - CD. In this way, the research questions were elaborated: what is the scientific evidence of educational interventions conducted by HP on CD in early childhood in a community context? What assumptions of HL are present in the implementation of these educational interventions?

### Sampling

For the selection of articles, three databases were used: Cumulative Index to Nursing and Allied Health Literature (CINAHL), Medical Literature Analysis and Retrieval System Online (MEDLINE) and Web of Science. All were accessed via the Journals Portal of the Coordination for the Improvement of Higher Education Personnel (CAPES - *Coordenação de Aperfeiçoamento de Pessoal de Nível Superior*), through the access provided by the *Universidade Federal de Pernambuco* (UFPE).

As a search strategy, controlled descriptors contained in the Health Sciences Descriptors (DeCS) and in the Medical Subject Headings (MeSH) were used. The terms were chosen according to the PICO strategy: P - “Health Personnel”; I - “Health Education”; C - “Education, Public Health Professional”; O - “Child Development”. During the search, they were combined with each other using the Boolean connectors AND or OR, as shown in Chart 1. Some particularities of the databases were taken into account, and in the MEDLINE database, the “clinical trial” filter was applied.

**Chart 1:** Search strategy used in databases, applied filters, publications found and selected, Brazil, 2021

DATABASE	SEARCH STRATEGY	FILTERS APPLIED	PUBLICATIONS FOUND (INITIAL SAMPLE)	SELECTED ARTICLES (FINAL SAMPLE)
MEDLINE	((“Health Education” [Mesh]) OR “Education, Public Health Professional” [Mesh])) AND “child Development” [MeSH]	- Clinical trial; - Period of last the 10 years (2012-2021).	83	8
Web of Science	((ALL=(“Health Education” OR “Education, Public Health Professional”)) AND ALL=(“Child Development”))	- Period of last the 10 years (2012-2021).	111	2
CINAHL	TX (“Health Education” OR “Education, Public Health Professional”) AND TX “Child Development” AND TX ( “Health Workers” OR “Health Personnel”)	- Period of last the 10 years (2012-2021).	106	1
TOTAL			300	11

We included original articles in Portuguese, English and Spanish that addressed the implementation of educational interventions conducted by HP on CD, in the period of the last 10 years (2012 to 2021), available in full text. We excluded theses, dissertations, literature review studies, protocols, studies focused on the mental illness of parents/caregivers, and studies with children with a disability, risk or delay in CD.

**Figure 1:**
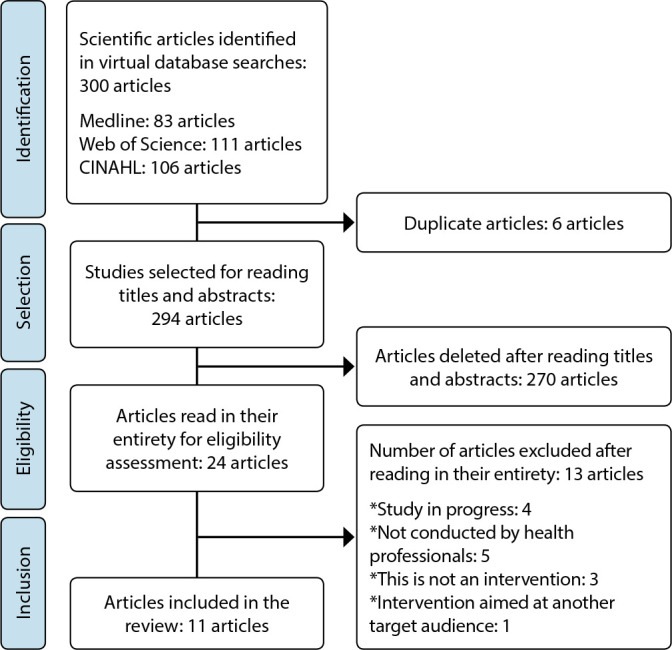
Study selection flowchart in the integrative review by PRISMA, Brazil, 2021

The search was performed under the Preferred Reporting Items for Systematic Reviews and Meta-Analyses (PRISMA) recommendations, presented in Figure 1. The search took place from September to October 2021 by two independent reviewers. A total of 300 studies were identified in the researched databases, and 11 were selected for the final sample. During selection, title and abstract were read, and when it was not discarded, the full text was read, verifying the pre-established eligibility criteria, including the exclusion of duplicate articles. Reference management software was not used. The articles were placed in a Microsoft Word spreadsheet for selection, reading, organization and categorization.

The steps of selection, reading, organization and categorization by the two reviewers were carried out independently, with periodic meetings for comparison and consensus in cases of divergence.

### Study categorization

To establish the level of evidence of the articles, the following classification was adopted: level I - meta-analyses and controlled and randomized studies; level II - experimental studies; level III - quasi-experimental studies; level IV - descriptive, non-experimental or qualitative studies; level V experience reports; and level VI - consensus and expert opinions^(11)^.

Extracted data were collected using an instrument developed by the author with the following items: study identification; year of publication; country; journal; objective; theoretical framework; educational material used in the intervention; children’s age group; target audience (parent/caregiver or other members of the child’s support network); and ending on CD.

Data were categorized, taking into account the HL domains, considering both educational interventions with HP and the direct application of knowledge and skills with caregivers, with the aim of improving indicators related to CD.

Data analysis was performed jointly between the reviewers and the advisor.

### Interpretation of results

Through a careful reading, the synthesis and descriptive and detailed interpretation of the findings were carried out. In the HL domains, Sorensen dimensions^(7)^ - access, understand, apply and appraise - were taken into account.

### Ethical and legal aspects

As this is a review study, collected from published articles and available in the literature, it is allowed to be carried out without the need for submission to Research Ethics Committee.

## RESULTS

### Study characterization

We included 11 articles (Chart 2) of intervention that met the eligibility criteria, all in English. We found 300 articles in the MEDLINE (83; 27.67%), Web of Science (111; 37%) and CINAHL (106; 35.33%) databases. After selection, eight MEDLINE articles were included, two from Web of Science and one from CINAHL.

**Chart 2:** Distribution of review articles according to database, year of publication, country, article title and level of evidence, Brazil, 2021

Study code/database	Year/ country	Article title	Level of evidence
A1: MEDLINE	2021/ Kenya	Group-based parenting interventions to promote child development in rural Kenya: a multi-arm, cluster-randomised community effectiveness trial^(12)^	I
A2: MEDLINE	2020/ China	The Effectiveness and Cost-effectiveness of a Parenting Intervention Integrated with Primary Health Care on Early Childhood Development: a Cluster-Randomized Controlled Trial^(13)^	I
A3: MEDLINE	2020/ Turkey	Effects of providing nursing care with web-based program on maternal self-efficacy and infant health^(14)^	I
A4: CINAHL	2020/ United States	Encouraging Parenting Behaviors That Promote Early Childhood Development Among Caregivers From Low-Income Urban Communities: A Randomized Static Group Comparison Trial of a Primary Care-Based Parenting Program^(15)^	I
A5: MEDLINE	2019/ China	Using community health workers to deliver a scalable integrated parenting program in rural China: A cluster-randomized controlled trial^(16)^	I
A6: MEDLINE	2018/ China	Effects of early comprehensive interventions on child neurodevelopment in poor rural areas of China: a moderated mediation analysis^(17)^	IV
A7: MEDLINE	2018/ Uganda	Nutrition, hygiene, and stimulation education to improve growth, cognitive, language, and motor development among infants in Uganda: A cluster-randomized Trial^(18)^	I
A8: Web of Science	2018/ United States	Comics as a Medium for Parent Health Education: Improving Understanding of Normal 9-Month-Old Developmental Milestones^(19)^	III
A9: MEDLINE	2016/ Pakistan	Effects of responsive stimulation and nutrition interventions on children’s development and growth at age 4 years in a disadvantaged population in Pakistan: a longitudinal follow-up of a cluster-randomised factorial effectiveness trial^(20)^	I
A10: MEDLINE	2014/ Pakistan	Effect of integrated responsive stimulation and nutrition interventions in the Lady Health Worker programme in Pakistan on child development, growth, and health outcomes: a cluster-randomised factorial effectiveness Trial^(21)^	I
A11: Web of Science	2012/ Banglandesh	Effects of a community-based approach of food and psychosocial stimulation on growth and development of severely malnourished children in Bangladesh: a randomized trial^(22)^	I

Regarding the time frame, the publications dated from 2012 to 2021, with an intensification of publications in the last five years, comprising 72.73% of the publications. Regarding the level of evidence, nine (81.82%) articles were randomized controlled trials (RCTs), with level of evidence I; one (9.09%) was a quasi-experimental study, with level of evidence III; and one (9.09%) was a descriptive study, with level of evidence IV. It is emphasized that the quasi-experimental study used randomization in its methods.

Regarding the origin of the interventions, there is a predominance in community and disadvantaged environments, low- and middle-income developing countries. Countries commonly found were China, Pakistan and the United States, accounting for more than 50% of publications. Other countries had been identified only once, such as Kenya, Turkey, Uganda and Banglandesh.

The rural area was the focus of six articles (A1, A5, A6, A7, A9 and A10), and the urban area, of three studies (A2, A4, A11). Although A11 was implemented in the urban area, it was intended specifically for slum dwellers, located in vulnerable areas, with inadequate facilities and environmental risk.

The age range of children at the time of enrollment in the intervention ranged from 0 to 36 months with follow-up reaching 48 months. Only in A3, children were enrolled prior to their birth. The final target audience worked was mainly mothers, followed by other caregivers that included parents and grandparents. Evidence showed positive outcomes in CD of children who received the intervention. Only A4 and A8 did not measure outcomes in CD.

Most of the interventions preceded with a training for HP before the performance of these with the final public. The HP training and assessment time took place before, during or after the implementation of the training. Only one article (A2) did not mention whether there was training of professionals. Of the eight articles that report the training, two (A7 and A11) did not describe how this stage occurred. In A3 and A8, the strategy was developed and implemented by the HP themselves, not performing a training.

Community health workers (CHW) applied the interventions (A1, A4 and A5) most frequently, followed by lady health workers (A9 and A10), nurses (A2 and A3), pediatricians and CD specialists (A2) and nutritionists (A7). A8 had a multidisciplinary team, composed of a doctor, nurse and others outside the health area (graphic designer). A2 and A11 mentioned that the implementation was performed by HP, but did not identify the class of professionals.

Regarding the use of a theoretical framework to support the interventions, two frameworks were identified: Albert Bandura’s Social Learning Theory (A4, A7 and A8) and Pender’s Health Promotion Model (A3). The other articles did not report theoretical frameworks for the basis of educational interventions.

### Theoretical framework in health literacy

Regarding the analysis and identification of the HL assumptions in the selected articles, as shown in Chart 3, it was divided according to the HL theoretical framework assumptions in accessing, understanding, appraising and applying, in order to identify them during the implementation of interventions.

**Chart 3:** Health literacy assumptions identified in interventions conducted by health professionals, Brazil, 2021

Study code	HEALTH LITERACY ASSUMPTIONS			
	Access	Understand	Appraise	Apply
A1: MEDLINE	Teaching-learning practices: group work, demonstration, problem-solving. Location: home, community centers or churches. Themes: play, responsive communication, hygiene, nutrition, love and respect.	Self-report from parents.	Self-report of positive appraisal.	Self-report of parental stimulation practices and qualitative interviews.
A2: MEDLINE	**Teaching-learning practices:** pamphlet and telephone call for parents of children with late signs. **Location:** childcare. **Themes:** games and activities to stimulate their children in skill development.	No information.	No information.	No information.
A3: MEDLINE	**Teaching-learning practices:** site prepared by nurses for primiparous mothers. Videos, motivational messages, possible situations that mothers may come across and how to solve them, in addition to a tab to ask questions of mothers to nurses through the site itself. **Location:** digital. **Themes:** nutrition, bathing and care, sleep and safety, and communication with the baby.	Knowledge appraisal with open-ended questions.	No information.	Form before and after the intervention.
A4: CINAHL	**Teaching-learning practices:** demonstration of examples for using age-specific toys and interactions with children. Feedbacks, with praise and reinforcement of positive behaviors. **Location:** waiting room and after childcare consultation. **Themes:** a toy was given to each parent along with a handout with suggestions for recreational activities. Comparison with control group (Centers for Disease Control and Prevention leaflet - CPD 9-Month-Old Developmental Milestones).	No information.	No information.	Self-report of parents, telephone questions about cognitive interactions with children and scales of self-efficacy and confidence in raising children.
A5: MEDLINE	**Teaching-learning practices:** CHWs trained caregivers in interactive activities with their children and provided guidance on child nutrition. **Location:** waiting room and, after childcare consultation, home. **Themes:** CD, food, immunization, hygiene, sleep, among others.	No information.	Telephone interviews so that caregivers can give their feedback on the quality of home visits.	Questionnaire to collect measures of parenting practices and parental beliefs, health promotion and measures of health-promoting eating practices.
A6: MEDLINE	**Practical of teach-learning:** check-up of child health, monitoring of CD, guidance on food and advice to develop recreational activities to encourage children’s learning. **Location:** home. **Themes:** food, development, recreational activities for stimulation and forms of communication.	No information.	No information.	Infant-Toddler Home Observation for Measurement of the Environment (IT-HOME).
A7: MEDLINE	**Teaching-learning practices:** providing immediate information and demonstrations (practice) of how to play with children. **Location:** home. **Themes: CD** (cognition, language, songs, gross motor and fine motor).	Questionnaire for knowledge appraisal.	No information.	No information.
A8: Web of Science	**Teaching-learning practices:** comic books. **Location:** childcare. **Themes:** CD milestones.	Pre- and post-intervention questionnaire, with eight questions aimed at understanding the CD milestones at 9 months in relation to children’s motor, language and social skills.	No information.	Parents reported the use of information obtained through the comic book; told a friend about CD; called the pediatrician to discuss possible delays in their child; and shared the material with relatives.
A9: MEDLINE	**Teaching-learning practices:** home visits. **Location:** home. **Themes:** responsive stimulation, CD and food.	Maternal self-report.	No information.	Observation of Mother-Child Interaction (OMCI) measure.
A10: MEDLINE	**Teaching-learning practices:** home visits, feedback. **Location:** home. **Themes:** feeding (control group), responsive stimulation, CD promotion (intervention group).	No information.	No information.	No information.
A11: Web of Science	**Teaching-learning practices:** five groups to receive intervention: (1) psychosocial stimulation (PS); (2) food supplementation (FS); (3) PS + FS; (4) clinical control (CC); and (5) hospital control (HC). **Location:** community clinics, home. **Themes:** toys, readings, CD milestones, nutritional supplementation.	No information.	No information.	No information.

In the “access” skill, the interventions used home visits (HV) in seven articles (A1, A5, A6, A7, A9, A10, A11) as strategies to provide the interventions, and group meetings (GM) in four articles (A1, A7, A9 and A10), childcare consultations in two articles (A2 and A6). Less frequently, other modalities were identified, such as the waiting room (A4) and parental pamphlet (A2). In another article (A3), the digital medium was used, with the provision of a website for guidance on child care, conducted by nurses. And an article (A8) delivered a comic book (CB). The themes most present in the interventions were psychosocial stimulation, playful activities between parents and children, nutritional education and general child care, including hygiene, safety and food.

As for the “understand” skill, not all studies measured parents’ understanding to ascertain whether the information had really been apprehended, only five articles (A1, A3, A7, A8, A9) carried out knowledge measurement or appraisal.

When analyzing the “appraise” skill, a gap was noticed in the studies. In A1, subjectively, the mothers reported having benefited from the intervention, and in A5, interviews were carried out to obtain feedback about the quality of the intervention.

The “apply” skill during the interventions could be observed, as HP identified and/or measured parenting attitudes and practices, using questionnaires or observations of the interaction between caregiver and child.

Table 1 shows the CD domains and the impacts achieved through interventions, whether positive (+), negative (-), or not significant (=). Some studies worked on all domains, while others worked only one (A4, A11) or three CD domains (A1, A2). By associating the CD domains with the HL assumptions, it is possible to observe a relationship between the outcomes achieved in children’s development and the presence of HL assumptions in the interventions implemented in each study.

**Table 1:** Description of studies according to child development outcome and domain of health literacy assumptions, Brazil, 2021

Article	Cognition/personal-social	Language	Socioemotional	Fine motor	Gross motor	Health literacy
E1	✓(+)	✓(+)	✓(+)			(1)(2)(3)(4)
E2		✓(+)	✓ (=)	✓(+)		(1)
E3	✓	✓	✓	✓	✓	(1)(2)(4)
E4	✓					(1)(4)
E5	✓(+)	✓(=)	✓(=)	✓(=)	✓(=)	(1)(3)(4)
E6	✓(+)	✓(=)	✓(+)	✓(+)	✓(=)	(1)(4)
E7	✓(+)	✓(+)	✓(=)	✓(+)	✓(=)	(1)(2)
E8						(1)(2)(4)
E9	✓(+)	✓(+)	✓(+)	✓(+)	✓(+)	(1)(2)(4)
E10	✓(+)	✓(+)	✓(+)	✓(+)	✓(+)	(1)
E11					✓(=)	(1)

*Note: ✓it was a domain of intervention; (+) the intervention had a positive impact on that domain; (-) the intervention had a negative impact on that domain; (=) the intervention had no significant impact on that domain; (1) access; (2) understand; (3) appraise; (4) apply.*

In A3 and A4, despite having worked on the CD domains, they did not bring the impact regarding the outcome achieved. In A3, it is reported that the development of the intervention group remained unchanged, and in A4, parent-child activities had a positive impact on parents’ playing and teaching activities, which are, in turn, closely associated with cognitive development promotion.

## DISCUSSION

The analysis of the evidence made it possible to verify different proposals for educational interventions, which consisted of two stages, the theoretical-practical training of HP on CD, followed by the application of knowledge and skills with caregivers.

The trend towards strengthening the health workforce to implement community educational interventions in low- and middle-income countries may be linked to the low educational level of caregivers of young children and the risks for them to reach their full development, and the interventions are mainly aimed at children from zero to three years old.

The educational frameworks used in the interventions were not explored in most studies. Only a few studies cited the Albert Bandura’s Theory of Social Learning (A4, A7 and A8) and Pender’s Health Promotion Model (A3) frameworks.

The adopted frameworks contribute to the importance of motivational and practical aspects in decision-making and behavioral choices, which are similar to HL propositions.

Teaching-learning strategies took place in environments such as waiting rooms, offices or homes, virtual environments, with emphasis on HV as a strategy that supports child/family’s individual needs.

The main themes explored are in line with the Nurturing Care Framework: health, nutrition and responsive care such as play, affection and games.

HL skills were present in the educational interventions conducted by HP on CD in early childhood in a community context, even if the HL framework was not used. PHC is a promising environment for the implementation of CD interventions, as it follows the children during the first years of life, presents an adequate infrastructure that enables the implementation of interventions and a non-stigmatizing location^(23)^.

Mothers were the main mediators for actions to promote their children’s development, followed by fathers and other caregivers in the child’s support network. Strategies were aimed at changing the behavior of parents/caregivers so that they were sensitive and responsive to children; this includes understanding their children’s signals and responding appropriately to their development. Stimulation involved playful activities, readings, games, playthings, music, among other activities involving parents and children, proving to be beneficial for CD^(12,15-17,20-22,24)^.

HP have been an important bridge in the delivery of CD-facing interventions with parents/caregivers, through childcare consultations^(13,17)^, HV^(12,16,18,20-22)^, GM^(12,18,20-21)^, waiting rooms^(13)^ or by means of other tools such as CB^(19)^ and website^(14)^. It is noticed that CHWS were the most involved in the interventions, highlighting their importance in the context of PHC and in child health surveillance^(12,15-16)^.

HP training is shown to be a necessary step to strengthen the implementation of the intervention, as it provides the proper preparation of professionals and the availability of materials for conducting activities with parents/caregivers^(12,16)^. Moreover, HP’s awareness of guiding and demonstrating to parents/caregivers playful and interactive activities conducive to CD is highlighted, as well as encouraging and praising parents’ resourcefulness in the care provided during the intervention.

The HL theoretical framework assumptions were identified in the interventions, as the interventions provided a means of accessing information, reaching the first skill and measured parents’ knowledge and attitudes/practices, reaching the second and fourth skills, respectively. The third skill was considered, even less frequently, with regard to the appraisal of the information obtained.

Obtaining information in the health area depends on several paths, and the first one refers to access to information. According to Sorensene et al.^(7)^, “access refers to the ability to search, find and obtain health information”. This process leads health users to obtain information that is focused on health promotion, prevention and domain.

In the interventions, two modalities of access for parents were investigated: (1) oral; (2) written and visual. The oral modality occurred through verbalized guidelines by HP about the necessary stimuli for children at each stage of their development, except for the telephone call, which was made only for parents whose children were flagged with a possible developmental delay^(13)^.

In Brazil, HV and childcare consultations are practices already established by the Ministry of Health (MoH) to be performed by HP within the PHC scope. At least one HV is recommended for the mother-baby dyad in the first week after birth, with seven childcare consultations being recommended in the first year of life. These are opportune moments to introduce educational strategies to guide parents about caring for their children and promoting CD^(25)^.

GM have been sufficiently being explored in the implementation of interventions. One of the benefits observed during its application is peer learning, which possibly occurs through the behavioral imitation of colleagues of similar ages, through the exchange of knowledge and sharing of experiences^(23)^. A similar hypothesis was raised in another study, by listing that mothers and children in groups learned skills through observation of other members^(26)^.

The written modality combined with the visual one was arranged in three ways: delivery of a CB about the CD milestones at nine months of age^(19)^, delivery of a parental pamphlet ^(13)^ and providing a website on child care^(14)^.

The educational technologies (ET) displayed in the aforementioned studies are tools used to mediate the educational process, corresponding to a resource that helps people’s way of learning. Despite the smaller amount of studies, it has been an area that has been gaining ground in HE and contributing to educational interventions in an effective way^(27)^.

Currently, technologies such as the internet and cell phones are more present and accessible in people’s daily lives, regardless of social class. These technological means enable communication between HP and parents, and it is a tool that enables the delivery of interventions. However, it is necessary to look at the existing disparities, mainly related to parents’ HL, which make the use of these technologies unequal. Parents who have a higher level of HL use it more frequently to obtain health information and manage their health, unlike those with a lower level of HL^(28)^.

After accessing the content, the skill to be achieved is understanding. Understanding health information consists of a person’s ability to understand the content accessed, involving a complex process in the interaction of each person’s own elements, such as logic, culture, language, among other factors that influence understanding and, consequently, better decision-making^(7)^.

Furthermore, measurement in reference to the educational intervention is not enough. To increase understanding, it is also important to develop educational messages aimed at improving the HL of these parents, improving their oral and written communication, since a higher level of HL will be directly related to positive health outcomes for their children^(6,29)^.

The use of images associated with written information, such as the elaboration of CB, can be an effective alternative to obtain greater understanding with parents with low education. This tool allows the transmission of complex messages in a simpler way, allowing that, through the association between the message and the image, the readers have a learning process and behavioral changes^(19)^. In that study, a pre- and post-intervention questionnaire was applied in the delivery of the CB, observing a significant increase in correct answers about CD after application of the intervention.

Another important aspect in HL concerns the “appraise” skill, which consists of a person’s ability to judge an intervention, information or guidance. “Appraise” includes a critical step, in which the person is able to verify the content accessed^(7)^.

This domain allows a person to identify whether the information accessed; in this case, if the educational intervention or other types of health guidelines are reliable and if they should be applied to manage their health or that of their family members. In this regard, no information was found in the studies that addressed this skill in a direct and specific way, with a gap identified, although it is believed that caregivers with more knowledge and skills are able to assess, on a daily basis, information from common sense for better decision-making.

In the intervention implemented by Luo et al.^(16)^, telephone interviews were mentioned so that caregivers could give their feedback on the quality of HV and, based on that, improve the conduct of interventions. However, the involvement and active participation of users in assessment processes are important, in which they can verify the consistency and applicability of the accessed and discussed knowledge. It is believed that mixed studies, which include a quantitative and qualitative approach, would be useful to gain greater insight into this dimension.

Application, in turn, is the operational and practical part, with the person’s ability to use the information obtained to make the best decision in health^(7)^; in the case of the articles investigated, to apply the information on CD in the daily care of their children.

To assess the “apply” skill, different approaches were identified in the analyzed studies, through parental reports, use of scales, application of questionnaires/forms or practical assessment instruments, in addition to direct observation. These means proved to be appropriate for measuring HP to achieve the intervention’s objectives, if they were being put into practice in the family context between parents/caregivers with their child or if there was a need for other stimuli for greater development potential. Moreover, it was an important tool for improving the next approaches/interventions.

By associating the outcomes achieved in the development of children included in the studies and the use of HL assumptions in interventions, a relationship between the HL assumptions and positive results in CD is noted. In A1, all assumptions were identified and all outcomes improved in a significantly positive way, while in A11, an assumption was addressed, and the outcome in CD did not improve significantly. Interventions covering the nuances of HL were reaffirmed as important and promising: access, understand, appraise and apply.

### Study limitations

Some limitations in the present review refer to the inclusion and exclusion criteria, such as the delimitation of languages and time frame and limitation in the amount of database used, which represents only a portion of this universe. We suggest that further studies be carried out with a greater scope on the subject in the future.

### Contributions to nursing, health, and public policies

The integrative review helps HP to reflect on higher education and assistance with parents/caregivers of children in early childhood. The relevance of diverse educational materials that can mediate effective communication between HP and caregivers is confirmed. Another highlight is the need for theoretical-practical training with HP so that they are agents of change in direct action with caregivers and families and achievement of results. The HL framework can support training and higher education processes in a clearer and more effective way.

## CONCLUSIONS

Educational interventions with professionals working in PHC should add access to up-to-date knowledge and the development of communicative skills and abilities to establish dialogic relationships based on the active listening of parents and caregivers of children, establishing interactive scenarios between parents/caregivers and children for affective and positive playful relationships for comprehensive development of children in early childhood, with the offer of recreational resources and even food nutrients that mark situations of exclusion and social invisibility.

The results point to the importance of training professionals on CD in early childhood in community contexts and, later, the application of knowledge and skills with caregivers, in order to exercise daily practices.

There is a limitation in the studies analyzed regarding the scarcity in the use of theoretical framework to support educational interventions. We emphasize the relevance of critical and problematizing theoretical frameworks for the basis and conduction of educational actions, highlighting here HL, for integrating skills and abilities for an effective and understandable communication in its constructs.
